# Design and Synthesis of Biomedical Polymer Materials

**DOI:** 10.3390/ijms25105088

**Published:** 2024-05-07

**Authors:** Jie Chen

**Affiliations:** Laboratory of Polymer Ecomaterials, Changchun Institute of Applied Chemistry, Chinese Academy of Sciences, Changchun 130022, China; chenjie@ciac.ac.cn

Due to their biocompatibility and non-toxic nature, biomedical polymer materials have found widespread applications and significantly propelled the progress of the biomedical field [1]. The advancing tide of biotechnology and medical technology has raised the bar for the quality and performance of biomedical materials. The design and synthesis of polymer materials stand as the cornerstone of their biomedical utilization. A diverse array of polymer materials, including nanoparticles, hydrogels, stimuli-responsive materials, and implant materials, have been subjected to rigorous investigation for their biomedical applications. Furthermore, polymer materials have found their niches in numerous biomedical application fields, ranging from tissue engineering and gene/drug delivery to medical device, diagnostics, antiviral, and antibacterial applications. In this Special Issue, we shed light on the intricate design, synthesis, functionalization, and prospects of polymer materials for biomedical applications [2].

In the realm of biomedicine, hydrogel has been widely applied due to its exceptional biocompatibility, remarkable water absorption and retention abilities, and resemblance to the extracellular matrix of tissue cells [3]. Wang and colleagues (Contribution 1) constructed a thermosensitive hydrogel by physically blending chitosan, sodium alginate, and β-glycerophosphate (β-GP). At ambient temperature, the CS/β-GP/SA mixture exhibits pristine clarity, appearing as a transparent solution. However, when exposed to 37 °C for 3 min, it transforms seamlessly into a uniform gel, a property that greatly enhances its potential for further biomedical applications. This temperature-responsive hydrogel serves as a harmless and nontoxic vehicle, adept at efficiently encapsulating and releasing berberine drugs. This berberine-loaded thermosensitive hydrogel is a potent therapeutic agent for treating periodontitis. It exerts anti-inflammatory and osteogenic effects by meticulously regulating the phosphatidylinositol-3 kinase/protein kinase B (PI3K/AKT) signaling pathway, offering a promising treatment option for periodontal health. Furthermore, the authors successfully crafted another thermosensitive hydrogel by meticulously blending CS, β-GP, and gelatin (Contribution 2). Notably, the administration of this temperature-sensitive hydrogel for local treatment within the body does not necessitate invasive surgical procedures. Instead, this hydrogel can be precisely injected into the targeted lesion site using a syringe, thereby greatly reducing surgical trauma and enhancing patient comfort. This hydrogel system also boasts the ability to achieve sustained drug release, effectively addressing the issues of low bioavailability and short half-life associated with IL-1ra. Additionally, the observed decrease in blood glucose levels in rats may be attributed to this hydrogel’s ability to effectively alleviate local periodontal inflammation, ultimately influencing overall blood glucose levels.

Polymer materials are prominent choices for wound dressings due to their unique properties and versatility. These materials offer a comprehensive solution for wound dressings, providing a biocompatible, absorbent, strong, flexible, and breathable barrier that promotes rapid and effective healing. With their versatility and adaptability, polymers are poised to continue playing a pivotal role in advancing wound care technologies [4]. Bankosz prepared a hydrogel dressing based on CS, gelatin, and albumin via the salt-induced protein precipitation process (Contribution 3). This hydrogel exhibits the ability to effectively release albumin in an acidic environment similar to neoplastic tissue, thus demonstrating a potential application of these hydrogels as materials that can support skin cancer treatment or injured tissue repair. Cai et al. (Contribution 4) designed and synthesized poly(g-benzyl-L-glutamate) (PBLG)-modified GdPO_4_·H_2_O nanobunches via hydroxylation, silylation, and glutamylation processes. GdPO_4_·H_2_O@SiO_2_ was further constructed by coating the surface of GdPO_4_·H_2_O with Si-OH. GdPO_4_·H_2_O@SiO_2_ exhibits good biocompatibility and enhances the expression of biomacromolecules such as COL I and COL II, making it a potential contributor in the fields of biomedical materials and tissue engineering. 

Nucleic acid is a unique biological macromolecule polymer, serving as an integral and vital component within organisms. It plays pivotal roles in the safeguarding and dissemination of genetic information, alongside orchestrating the intricate processes of biosynthesis [5]. Gan and colleagues (Contribution 5) prepared highly compressed DNA nanoflowers with prolonged native DNA backbones and repeated inhibitory oligodeoxynucleotides (INH-ODN) that can exert an immunomodulatory effect to specifically block TLR7 and TLR9 signaling in systemic lupus erythematosus (SLE). The nanoflowers, as endogenous ligands of TLR7 and TLR9, contribute to decreasing autoantibody levels, reducing cytokine secretion, and alleviating SLE in mice and provide a new strategy for exploring lupus therapeutic agents. The applications of macromolecular nucleic acids in biomedicine are diverse and hold tremendous potential. With further research and development, these molecules are expected to play an increasingly important role in improving human health and treating diseases.

Asthma, a chronic inflammatory disease that affects the airways, is a prevalent disease among humans. It manifests in recurrent episodes of coughing, wheezing, and difficulty breathing, which seriously affect individuals’ longevity and quality of life. Nanotechnology, an emerging tool, has garnered extensive research attention in the field of asthma. Zuo et al. delve into the intricate mechanisms underlying asthma development and the current therapeutic approaches employed (Contribution 6). Furthermore, they present a comprehensive overview of the design and synthesis of diverse nanomaterials and micromaterials designed to treat asthma. These materials range from polymeric nanomaterials to solid lipid nanomaterials, cell-membrane-based nanomaterials, and metal nanomaterials. Lastly, the authors discuss the challenges and prospects of these nanomaterials, offering valuable insights for future research directions and potentially propelling the clinical application of nanotherapeutics in asthma treatment.

Poly(lactic-co-glycolic acid) (PLGA) is a biodegradable and biocompatible polymer that is significantly important in the field of biomedicine. Its unique properties allow its widespread utilization in drug delivery systems, tissue engineering, and regenerative medicine [6]. PLGA can be customized to regulate the release rates of encapsulated drugs, thereby providing sustained therapeutic benefits [7]. Furthermore, its biodegradability ensures safe metabolism and elimination from the body, minimizing the risk of potential adverse effects [8]. Consequently, PLGA plays a pivotal role in enhancing the efficacy and safety of biomedical applications. Guo et al. (Contribution 7) conducted a comprehensive review of the synthesis and modification methods of PLGA M/NPs, summarizing their application advancements in the treatment of respiratory diseases over the past few years. Finally, the authors comprehensively summarize the application of PLGA M/NPs in the biomedical field, along with its development prospects, potential obstacles, and proposed solutions. 

This compilation of articles is devoted to exploring the design, synthesis, and application of biomedical polymer materials. Undoubtedly, this Special Issue is poised to captivate the attention of researchers across various domains, encompassing materials science, biomedical research, and even clinical practices. However, it is worth noting that the realm of biomedical polymer materials is vast and diverse, with applications spanning a breadth of which this Special Issue can only scrape a small part. Biomedical polymer materials primarily consist of organic compounds, offering the remarkable flexibility of allowing their biocompatibility to be tailored by adjusting their molecular structures. Some of them even exhibit a striking resemblance to human tissues in terms of morphology and properties, making them compatible with living organisms and capable of functioning harmlessly within the human body. These materials possess a remarkable softness that allows them to adapt to diverse shapes and sizes of biological organs, allowing them to effortlessly navigate through the human body. This adaptability has led to numerous applications of medical polymer materials, including drug delivery, bioimaging, prostheses, stents, and sutures [9]. Furthermore, these materials can be enhanced with a range of functional components, such as antibacterial agents and growth factors, to fulfill specific physical, chemical, and biological functions. By incorporating these specialized functionalities, these materials offer immense promise and have emerged as invaluable tools in medical research and clinical treatment. However, despite their promising initial performance, there remain challenges in the application of medical polymer materials [10]. Some researchers have pointed out that the long-term stability, biocompatibility, and controllability of these materials still require rigorous research and verification to meet the demands of clinical applications.

Future research on biomedical polymer materials is expected to lead to the development of new and improved materials with enhanced biocompatibility, functionality, and degradability. This path may encompass the disclosure of innovative polymers, the alteration of current materials, or the creation of composite materials that offer a convergence of benefits. Despite the noteworthy challenges present in the domain of biomedical polymer materials, the outlook for forthcoming advancements and applications remains encouraging. The perseverance in research and innovation within this sphere is anticipated to lead to momentous advancements in patient care and the sustainability of healthcare systems.
